# Supplementary material for: Quantitative Proteomics Identification of Seminal Fluid Proteins in Male Drosophila melanogaster
Source: Mol Cell Proteomics. 2018 Oct 4;18(Suppl 1):S46–58. doi: 10.1074/mcp.RA118.000831 (PMC6427238; doi:10.1074/mcp.RA118.000831)
Supplement: Supplementary information [file 137877_1_supp_167585_pbr1x9.docx]

**Quantitative proteomics identification of seminal fluid proteins in male *Drosophila melanogaster***

Irem Sepil, Ben R Hopkins, Rebecca Dean, Marie-Laëtitia Thézénas, Philip D Charles, Rebecca Konietzny, Roman Fischer, Benedikt M Kessler, Stuart Wigby

**Supplementary Information**

**Table S1** **-** The list of the proteins that are identified as candidate Sfps in this study, a summary of the first set of criteria they met and their functional categories. These criteria were: **(1)** Significantly higher abundance (p ≤ 0.05) in unmated male samples (Male Dataset 1) and, if present, higher abundance in unmated male samples (Male Dataset 2); **(2)** Significantly higher abundance (p ≤ 0.05) in unmated male samples (Male Dataset 2) and, if present, higher abundance in unmated male samples (Male Dataset 1); **(3)** Presence of a signal peptide; **(4)** Exclusive expression in accessory glands. The proteins are ordered according to their functional categories. NA stands for no data available.

| **Protein name** | | **Criteria 1:  Higher in unmated  males (Dataset 1)** | **Criteria 2:  Higher in unmated  males (Dataset 2)** | **Criteria 3:  Presence of signal peptide** | **Criteria 4:  Accessory gland  exclusive expression** | **First set of criteria TOTAL** | **Functional categories** |
| --- | --- | --- | --- | --- | --- | --- | --- |
| Treh | 1 | 1 | 1 | 0 | 3 | Carbohydrate interactions |  |
| Fas1 | 1 | 1 | 1 | 0 | 3 | Cell adhesion |  |
| CG31413 | 1 | 1 | 1 | 1 | 4 | Cell redox homeostasis |  |
| Idgf3 | 1 | 1 | 1 | 0 | 3 | Chitin binding |  |
| Idgf4 | 1 | 1 | 1 | 0 | 3 | Chitin binding |  |
| sphinx2 | 1 | 1 | 1 | 1 | 4 | Defense / immunity |  |
| GLaz | 1 | 1 | 1 | 0 | 3 | Determination of adult lifespan |  |
| Adgf-C | 1 | 1 | 1 | 1 | 4 | DNA interactions |  |
| NT5E-2 | 1 | 1 | 1 | 0 | 3 | DNA interactions |  |
| CG9519 | 1 | 0 | 1 | 1 | 3 | Hormone metabolism |  |
| CG11600 | 1 | 0 | 1 | 1 | 3 | Lipid metabolism |  |
| CG18258 | 1 | 1 | 1 | 1 | 4 | Lipid metabolism |  |
| **Protein name** | **Criteria 1:  Higher in unmated  males (Dataset 1)** | **Criteria 2:  Higher in unmated  males (Dataset 2)** | **Criteria 3:  Presence of signal peptide** | **Criteria 4:  Accessory gland  exclusive expression** | **First set of criteria TOTAL** | **Functional categories** |  |
| CG31684 | 1 | 1 | 1 | 1 | 4 | Lipid metabolism |  |
| CG11590-RA | 1 | 0 | 1 | 1 | 3 | Metal ion binding |  |
| Obp8a | 1 | 1 | 1 | 1 | 4 | Odorant binding |  |
| CG43061 | 0 | 1 | 1 | 1 | 3 | Pathogenesis |  |
| CG11034 | 1 | 1 | 1 | 0 | 3 | Protease |  |
| CG11955 | 1 | 1 | 1 | 0 | 3 | Protease |  |
| CG2111 | 1 | 0 | 1 | 1 | 3 | Protease |  |
| CG3097 | 1 | 1 | 1 | 0 | 3 | Protease |  |
| CG34295 | 1 | 0 | 1 | 1 | 3 | Protease |  |
| CG40160 | 1 | 1 | 1 | 0 | 3 | Protease |  |
| CG6357 | 1 | 1 | 1 | 0 | 3 | Protease |  |
| CG9806 | 1 | 0 | 1 | 1 | 3 | Protease |  |
| CG42467 | 1 | 0 | 1 | 1 | 3 | Protease inhibitor |  |
| CG43145 | 1 | 1 | 1 | 1 | 4 | Protease inhibitor |  |
| Spn28Db | 1 | 1 | 0 | 1 | 3 | Protease inhibitor |  |
| Spn42De | 1 | 0 | 1 | 1 | 3 | Protease inhibitor |  |
| Spn88Ea | 1 | 1 | 1 | 0 | 3 | Protease inhibitor |  |
| Gld | 1 | 1 | 1 | 1 | 4 | Sperm storage |  |
| intr | 1 | 1 | 0 | 1 | 3 | Sperm storage / post-mating behaviour |  |
| Acp54A1 | 0 | 1 | 1 | 1 | 3 | Unknown function |  |
| BG642163 | 1 | 0 | 1 | 1 | 3 | Unknown function |  |
| CG11977 | 1 | 0 | 1 | 1 | 3 | Unknown function |  |
| CG12093 | 1 | 1 | 1 | 0 | 3 | Unknown function |  |
| CG12828-RA | 1 | 1 | 1 | 1 | 4 | Unknown function |  |
| CG15394 | 1 | 1 | 1 | 1 | 4 | Unknown function |  |
| CG17549 | 1 | 1 | 1 | 0 | 3 | Unknown function |  |
| CG33290-RA | 1 | 1 | 1 | 1 | 4 | Unknown function |  |
| CG3640 | 1 | 1 | 1 | 1 | 4 | Unknown function |  |
| CG42471 | 1 | 1 | 1 | 1 | 4 | Unknown function |  |
| CG42682 | 1 | 0 | 1 | 1 | 3 | Unknown function |  |
| CG42782 | 0 | 1 | 1 | 1 | 3 | Unknown function |  |
| **Protein name** | **Criteria 1:  Higher in unmated  males (Dataset 1)** | **Criteria 2:  Higher in unmated  males (Dataset 2)** | **Criteria 3:  Presence of signal peptide** | **Criteria 4:  Accessory gland  exclusive expression** | **First set of criteria TOTAL** | **Functional categories** |  |
| CG43057-RA | 1 | 0 | 1 | 1 | 3 | Unknown function |  |
| CG43101-RA | 0 | 1 | 1 | 1 | 3 | Unknown function |  |
| CG43111 | 0 | 1 | 1 | 1 | 3 | Unknown function |  |
| CG43123 | 0 | 1 | 1 | 1 | 3 | Unknown function |  |
| CG43147 | 0 | 1 | 1 | 1 | 3 | Unknown function |  |
| CG43307 | 1 | 0 | 1 | 1 | 3 | Unknown function |  |
| CG44574 | 1 | 1 | 1 | 1 | 4 | Unknown function |  |
| CG45011 | 1 | 0 | 1 | 1 | 3 | Unknown function |  |

**Table S2** **-** The functional annotation chart of the proteins that are significantly more abundant in virgin females against all the proteins identified in the female reproductive tract proteome (Female Dataset). Database for Visualization and Integrated Discovery (DAVID) was used for gene ontology (GO) enrichment analysis and the resulting p-values were Benjamini corrected for multiple testing. “Term” describes the enriched (overrepresented) annotation term; “Protein number” describes the number of proteins falling into this category (out of the 267 proteins); and “p-value” describes the multiple test corrected enrichment p-values. The terms are ordered according to their enrichment p-values.

| **Term** | **Protein number** | **p value** |
| --- | --- | --- |
| Immunoglobulin-like fold | 14 | 0.024 |
| Immunoglobulin-like domain | 10 | 0.034 |
| Immunoglobulin subtype | 9 | 0.043 |
| Immunoglobulin subtype 2 | 9 | 0.043 |
| Immunoglobulin I-set | 8 | 0.049 |

**Table S3** **-** The list of the previously identified Sfps that are confirmed in this study and a summary of the criteria they met to be classified as confirmed Sfps. These criteria were significantly higher abundance in unmated male samples (Male Dataset 1 or Male Dataset 2) and significantly higher abundance in mated female samples (Female Dataset). The proteins are ordered according to their gene names.

| **Protein** | **Criteria 1:  Higher in unmated  males (Dataset 1)** | **Criteria 2:  Higher in unmated  males (Dataset 2)** | **Criteria 5:  Higher in mated  females** |
| --- | --- | --- | --- |
| Acp26Aa | 1 | 1 | 1 |
| Acp26Ab | 1 | 0 | 0 |
| Acp29AB | 1 | 1 | 1 |
| Acp33A | 0 | 1 | 0 |
| Acp36DE | 1 | 1 | 1 |
| Acp53C14a | 1 | 1 | 1 |
| Acp53C14b | 1 | 1 | 1 |
| Acp53C14c | 1 | 1 | 1 |
| Acp53Ea | 1 | 1 | 1 |
| Acp62F | 1 | 1 | 1 |
| Acp63F | 0 | 1 | 1 |
| Acp76A | 1 | 0 | 0 |
| alphaTub84B | 0 | 0 | 1 |
| antr | 1 | 1 | 1 |
| aqrs | 1 | 1 | 1 |
| betaTub85D | 0 | 0 | 1 |
| BG642312 | 1 | 1 | 1 |
| BP1025 | 0 | 0 | 1 |
| CG10041 | 1 | 1 | 1 |
| CG10284 | 1 | 0 | 0 |
| CG10407 | 0 | 0 | 1 |
| CG10587 | 1 | 1 | 1 |
| CG10651 | 1 | 0 | 0 |
| CG10730 | 1 | 1 | 1 |
| CG11037 | 1 | 1 | 0 |
| CG11112 | 1 | 1 | 0 |
| CG11598 | 1 | 1 | 1 |
| CG11608 | 1 | 1 | 1 |
| CG14034 | 1 | 1 | 1 |
| CG15117 | 1 | 1 | 1 |
| CG15635 | 0 | 0 | 1 |
| CG15641 | 1 | 1 | 0 |
| CG1701 | 0 | 1 | 0 |
| CG17093 | 1 | 1 | 1 |
| CG17097 | 1 | 1 | 1 |
| CG17242 | 1 | 1 | 1 |
| CG17472 | 1 | 1 | 1 |
| CG17575 | 0 | 0 | 1 |
| CG17843 | 1 | 1 | 1 |
| CG17919 | 1 | 1 | 1 |
| CG18067 | 1 | 1 | 1 |
| **Protein** | **Criteria 1:  Higher in unmated  males (Dataset 1)** | **Criteria 2:  Higher in unmated  males (Dataset 2)** | **Criteria 5:  Higher in mated  females** |
| CG18135 | 0 | 0 | 1 |
| CG18284 | 1 | 1 | 1 |
| CG2852 | 1 | 1 | 1 |
| CG30395 | 1 | 1 | 1 |
| CG30486 | 1 | 0 | 0 |
| CG31418 | 1 | 1 | 1 |
| CG31419 | 1 | 1 | 1 |
| CG31515 | 1 | 1 | 0 |
| CG31659 | 1 | 1 | 1 |
| CG31680 | 1 | 1 | 1 |
| CG31704 | 1 | 1 | 0 |
| CG31883 | 1 | 1 | 1 |
| CG32833 | 1 | 0 | 1 |
| CG34002 | 1 | 1 | 0 |
| CG34033 | 1 | 1 | 1 |
| CG34034 | 1 | 1 | 0 |
| CG34051 | 1 | 0 | 1 |
| CG34129 | 1 | 1 | 0 |
| CG34130-RA | 1 | 1 | 1 |
| CG42564 | 0 | 1 | 1 |
| CG43319 | 0 | 1 | 0 |
| CG4847 | 1 | 0 | 0 |
| CG5162 | 1 | 1 | 1 |
| CG6071 | 1 | 1 | 1 |
| CG6690 | 1 | 1 | 1 |
| CG9029 | 1 | 0 | 0 |
| CG9168 | 1 | 0 | 1 |
| CG9997 | 1 | 1 | 1 |
| Cys | 0 | 1 | 0 |
| Dup99B | 1 | 1 | 1 |
| Ebp | 0 | 0 | 1 |
| EbpII | 0 | 0 | 1 |
| Est-6 | 1 | 1 | 1 |
| Ggt-1 | 1 | 1 | 1 |
| Hexo2 | 1 | 1 | 1 |
| lectin-29Ca | 1 | 1 | 1 |
| lectin-30A | 1 | 1 | 1 |
| lectin-46Ca | 1 | 1 | 1 |
| lectin-46Cb | 1 | 1 | 1 |
| Met75Ca | 1 | 1 | 1 |
| mfas | 1 | 0 | 1 |
| Mst57Da | 0 | 1 | 1 |
| Mst57Dc | 1 | 1 | 1 |
| NLaz | 1 | 0 | 0 |
| Npc2b | 1 | 0 | 1 |
| NUCB1 | 1 | 0 | 1 |
| Obp22a | 1 | 1 | 0 |
| Obp51a | 1 | 1 | 1 |
| Obp56e | 1 | 1 | 1 |
| **Protein** | **Criteria 1:  Higher in unmated  males (Dataset 1)** | **Criteria 2:  Higher in unmated  males (Dataset 2)** | **Criteria 5:  Higher in mated  females** |
| Obp56f | 1 | 1 | 1 |
| Obp56g | 1 | 0 | 1 |
| Obp56i | 1 | 1 | 1 |
| Phm | 1 | 1 | 0 |
| Porin2 | 0 | 0 | 1 |
| regucalcin | 1 | 1 | 1 |
| S-Lap7 | 0 | 0 | 1 |
| Semp1 | 1 | 1 | 0 |
| Sems | 1 | 1 | 1 |
| Sfp23F | 1 | 0 | 1 |
| Sfp24Ba | 0 | 0 | 1 |
| Sfp24Bb | 0 | 0 | 1 |
| Sfp24Bc | 0 | 0 | 1 |
| Sfp24Bd | 0 | 0 | 1 |
| Sfp24C1 | 1 | 1 | 1 |
| Sfp24F | 1 | 1 | 1 |
| Sfp26Ac | 0 | 1 | 1 |
| Sfp26Ad | 1 | 1 | 0 |
| Sfp33A3 | 1 | 1 | 1 |
| Sfp35C | 1 | 1 | 1 |
| Sfp38D | 1 | 1 | 1 |
| Sfp51E | 1 | 1 | 1 |
| Sfp65A | 1 | 1 | 1 |
| Sfp70A4 | 1 | 1 | 1 |
| Sfp78E | 1 | 1 | 0 |
| Sfp87B | 0 | 0 | 1 |
| SP | 1 | 1 | 0 |
| SP191 | 0 | 0 | 1 |
| Spn28B | 1 | 1 | 1 |
| Spn28F | 1 | 1 | 1 |
| Spn38F | 1 | 0 | 1 |
| Spn42Dd | 1 | 1 | 0 |
| Spn75F | 1 | 1 | 1 |
| Spn77Bb | 1 | 1 | 0 |
| Spn77Bc | 1 | 1 | 1 |

**Table S4** **-** The list of the putative ejaculatory duct specific proteins identified in this study, their functional categories and whether they have been linked to the *D. melanogaster* ejaculatory duct in previous studies.

| **Putative ejaculatory**  **Duct specific protein** | **Functional Category** | **Previously linked**  **to ejaculatory duct** |
| --- | --- | --- |
| CG17242 | Protease | no |
| CG18067 | Unknown function | no |
| CG31704 | Unknown function | no |
| CG34034 | Unknown function | yes (Takemori & Yamamoto 2009) |
| CG5162 | Lipid metabolism | yes (Takemori & Yamamoto 2009) |
| CG5402 | Unknown function | no |
| Dup99B | Post-mating behaviour | yes (Saudan et al. 2002) |
| Met75Ca | Unknown function | yes (Findlay et al. 2008) |
| Obp51a | Odorant binding | yes (Takemori & Yamamoto 2009) |
| Spn77Bb | Protease inhibitor | yes (Takemori & Yamamoto 2009) |
| Spn77Bc | Protease inhibitor | yes (Takemori & Yamamoto 2009) |

**Fig. S1 –** Experimental protocol for **(a)** male experiment 1, where male age and mating history was varied; **(b)** male experiment 2, where the males’ social environment was varied.

**Fig. S2 –** **Boxplot of the abundances of candidate ejaculatory duct specific proteins in accessory gland only samples (AG), ejaculatory duct only samples (DU) and samples containing both the ejaculatory duct and accessory gland (BO).** The 3 previouly known Sfps were significantly more abundant (p ≤ 0.05) in DU compared to AG. However they were similarly abundant in BO and AG samples.

**Data S1 –** The list of proteins identified in this study for Male Dataset 1, Male Dataset 2, Female Dataset and Male Tissue Dataset (table attached separately).

**Data S2** – The list of peptides identified in this study for Male Dataset 1, Male Dataset 2, Female Dataset and Male Tissue Dataset (table attached separately).

**Data S3** – The list of proteins that are significantly more abundant in mated females in comparison to virgin females that are likely to be female-derived. Known Sfps, high-confidence candidate Sfps and known sperm proteins and have been omitted from the list as these are male-derived proteins.
